# Activation of the renal GLP‐1R leads to expression of *Ren1* in the renal vascular tree

**DOI:** 10.1002/edm2.234

**Published:** 2021-03-19

**Authors:** Katrine Dahl Bjørnholm, Maria Elm Ougaard, Gry Freja Skovsted, Lotte Bjerre Knudsen, Charles Pyke

**Affiliations:** ^1^ Department of Experimental Animal Models University of Copenhagen Frederiksberg Denmark; ^2^ Department of Cardiovascular Research Novo Nordisk A/S Måløv Denmark; ^3^ Department of Pathology and Imaging Novo Nordisk A/S Måløv Denmark; ^4^ Diabetes Research Novo Nordisk A/S Måløv Denmark

**Keywords:** GLP‐1, GLP‐1 receptor, GLP‐1R agonists, in situ hybridization, renal vasculature, renin recruitment

## Abstract

The GLP‐1 receptor (GLP‐1R) in the kidney is expressed exclusively in vascular smooth muscle cells in arteries and arterioles. Downstream effects of the activation of the renal vascular GLP‐1R are elusive but may involve regulation of the renin‐angiotensin‐aldosterone system (RAAS). The expression of *Ren1* in the mouse renal vasculature was investigated by in situ hybridization after a single subcutaneous dose of liraglutide, semaglutide and after repeated injections of liraglutide. Single and repeated exposure to GLP‐1R agonists induced expression of *Ren1* in the renal vascular smooth muscle cell compartment compared with vehicle injected controls (*p* < .0001) for both semaglutide and liraglutide. The present data show a robust induction of *Ren1* expression in the vascular smooth muscle cells of the kidney after single and repeated GLP‐1R activation and this renin recruitment may be involved in the effects of GLP‐1R agonist treatment on kidney disease.

## Graphical abstract

Single and repeated injections with long acting GLP‐1R agonists leads to induction of Ren1 transcription from VSMC in renal vasculature in mice.

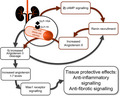

## 1 INTRODUCTION

GLP‐1 is a postprandial hormone released upon a carbohydrate rich meal to stimulate glucose dependent insulin secretion, decrease glucagon secretion, and gastric motility, and increase satiety.^1^ In patients, long‐acting GLP‐1R analogues such as liraglutide and semaglutide have been used for treatment of type 2 diabetes mellitus (T2DM), and obesity and are known to decrease cardiovascular disease and chronic kidney disease in people with T2DM.^2^, ^3^, ^4^, ^5^ The mechanism behind this has been studied in animal models and has been suggested to involve anti‐inflammatory properties,^6^ local and systemic haemodynamic effects,^7^ and natriuretic effects.^8^ It has also been suggested that the effect could involve regulation of RAAS.^9^ Interestingly, in humans the systemic blood levels of renin, angiotensin and aldosterone are only slightly or not affected by treatment with GLP‐1R agonists (GLP‐1RA).^10^, ^11^

In the kidney, the GLP‐1R is expressed exclusively in vascular smooth muscle cells (VSMC) of arteries and arterioles, and in the juxtaglomerular apparatus (JGA), including in specialized renin‐producing SMC cells located at the glomerular poles.^12^ Upon decrease in blood pressure, sympathetic stimulation or decrease in distal tubule sodium levels, the renin producing cells of the JGA secrete renin to the systemic circulation. Renin is an aspartyl protease with an active domain that binds angiotensinogen and converts it to angiotensin I, which is further cleaved by angiotensin converting enzyme to angiotensin II. Angiotensin II receptors are expressed in endothelial cells, VSMC, neuronal cells, adrenal glomerulosa and renal tubular cells. Stimulation of the angiotensin II receptor type‐1 (AT_1_) leads to sodium reabsorption, vasoconstriction, aldosterone secretion and sympathetic nerve activation, ultimately restoring blood pressure and kidney perfusion.^13^ A second angiotensin II receptor is present in renal endothelial cells leading to a nitric oxide dependent vasodilation opposing the effects of AT_1_ receptor stimulation.^14^

In humans, renin is coded for by *REN*, whereas in some mouse strains two genes code for Renin: *Ren1* and *Ren2*.^15^ The C57BL/6 strain only have one gene for renin: *Ren1*, which holds 76% homology to human *REN*.^16^ The *Ren1* gene codes for the renin precursor, the pro‐renin complex, which is cleaved into active renin by association with a soluble or membrane bound pro‐renin receptor.^17^ Besides activation of the pro‐renin complex, the membranous pro‐renin receptor is involved in intracellular signalling including regulation of cell survival, cell cycle, growth and intracellular acidosis.^18^ In rodents, transgenic manipulation of renin expression levels can lead to renal disease, hypertension and inflammation^19^ indicating that tight regulation of renin transcription and bioactivation could play an important role as a physiological regulator of kidney function. This study describes the effect of GLP‐1RAs on the expression of *Ren1* mRNA in the mouse kidney after acute and chronic exposure.

## 2 MATERIALS AND METHODS

### 2.1 In vivo

The studies were approved by the Danish Animal Experimentation Inspectorate under the Ministry of Environment and Food and carried out by trained and licensed personnel. All animals were housed in cages of eight animals per cage under 12‐h light dark cycle at 22 ± 2°C and 50 ± 20% humidity with ad libitum access to food and water. The mice were acclimatized for two weeks prior to study initiation. Animal caretakers inspected animals daily.

### 2.2 Experimental protocol—four hours exposure to semaglutide or liraglutide

Thirty C57BL/6J male mice, 8–10 weeks (Taconic, Lille Skensved, Denmark), were injected once subcutaneously with vehicle (pH = 7.4; 50 mmol/L phosphate; 70 mmol/L sodium chloride; 0.05% polysorbate 80, *n* = 10), semaglutide (15 nmol/kg, *n* = 10) or liraglutide (270 nmol/kg, *n* = 10), all from Novo Nordisk A/S, Denmark. Four hours after injection, animals were anaesthetized by inhalation of isoflurane, euthanized by cervical dislocation, and kidneys were collected.

### 2.3 Experimental protocol—26 days exposure to liraglutide in NTN mice

Eighteen C57BL/6J male mice, 8–10 weeks (Taconic, Lille Skensved Denmark), were divided into three groups. Two groups were anaesthetized in isoflurane and pre‐sensitized by two subcutaneous injections of 100 µg sheep IgG (P130‐100, lot#1128‐7, Bethyl Laboratories Inc, Denmark) in Complete Freund's adjuvant (5 mg/mL *mycobacterium butyricum* in mineral oil, MP Biomedicals, Eschwege, Germany) each injection. Four days later, 100µL nephrotoxic serum (NTS) from sheep was given intravenously in the tail vein (Probetex, San Antonio, USA), Batch PTX‐001S). One group received once daily subcutaneous injection with vehicle (pH = 7.4; 50 mmol/L phosphate; 70 mmol/L sodium chloride; 0.05% polysorbate 80) or liraglutide (270 nmol/kg, Novo Nordisk, Måløv, Denmark) after a dose titration scheme of 0.3, 0.6 and 1 mg/mL each day such that the animals received 1 mg/mL on the day of the NTS injection. The last group (*n* = 6) did not get NTS and was left untreated, thus serving as a naïve control. Following 26 days of treatment, animals were euthanized approximately 24 h after last dosing and the right kidney was collected.

### 2.4 In situ hybridization of REN1 and immunohistochemical staining of GLP1R

All kidneys were dissected free of surrounding tissue and placed in 10% neutral buffered formalin (NBF, 16004‐130, VWR), processed in the Leica ASP 300 S tissue processor (Leica Biosystems, Buffalo Grove, IL, USA) and embedded in paraffin for sectioning.

Kidneys were cut (4.5 µm) and mounted onto Superfrost Plus slides (Termo Fisher Scientific, Waltham, MA, USA) and placed in the Ventata Discovery XT automation system (Ventana Inc., Roche, Tucson, USA). The RNAscope technology was used to perform in situ hybridization using the VS 2,5 RED reagent kit (ref: 322220) and probe Mm‐Ren1 (ref: 322220). A section was hybridized with a probe for bacterial DapB (ref: 312039) and for PPIB (313919) serving as negative and positive control, respectively (all from Advanced Cell Diagnostics, Newark, CA). The slides were pre‐treated with protease and target retrieval. Some slides were double stained with Rb‐GLP‐1R antibody (2.7 µg/mL, ab218532 Abcam, Cambridge, UK) enhanced using the Rb‐HQ and a‐HQ‐HRP kits (760–4818 and 760–4820, respectively, Roche, Basel, Switzerland) and labelled using FITC in TSA (Roche, Basel, Switzerland). All slides were counterstained with DAPI for 8 minutes (10^−4^ µg/mL). Cover slides were mounted using DAKO fluorescent mounting media (DAKO, Glostrup, Denmark).

### 2.5 Quantification of histological staining

Slides were scanned using the Olympus VS120 Virtual Slide Microscope scanner (Olympus Corporation, Tokyo, Japan) and dots were quantified using the HALO software (Indica Labs, Albuquerque, USA) FISH algorithm version 2.1.7. A subset of arteries (3–10 per kidney dependent on availability on specific section) were selected. The muscularized artery should lie adjacent to a vein and free of perivascular adipose tissue. Results are shown as H‐score calculated, as recommended by manufacturer, from the equation: H = [1 × (cell fraction with 1–3 dots/cell) + 2 × (cell fraction with 4–9 dots/cell) + 3 × (cell fraction with 10–15 dots/cell) + 4 × (cell fraction with >15 dots/cell)].

### 2.6 Statistics

Statistical analyses were performed using GraphPad Prism version 8 (Graph Pad, San Diego, California). Differences with *p* < .05 were considered as significant. Individual values represent the mean of 3–9 arteries from the same mouse. All data are presented as individual values, mean and standard error of the mean (SEM).

## 3 RESULTS

### 3.1 Effect of 4 h of GLP‐1RA injection on Ren1 expression in naïve mice

**FIGURE 1:**
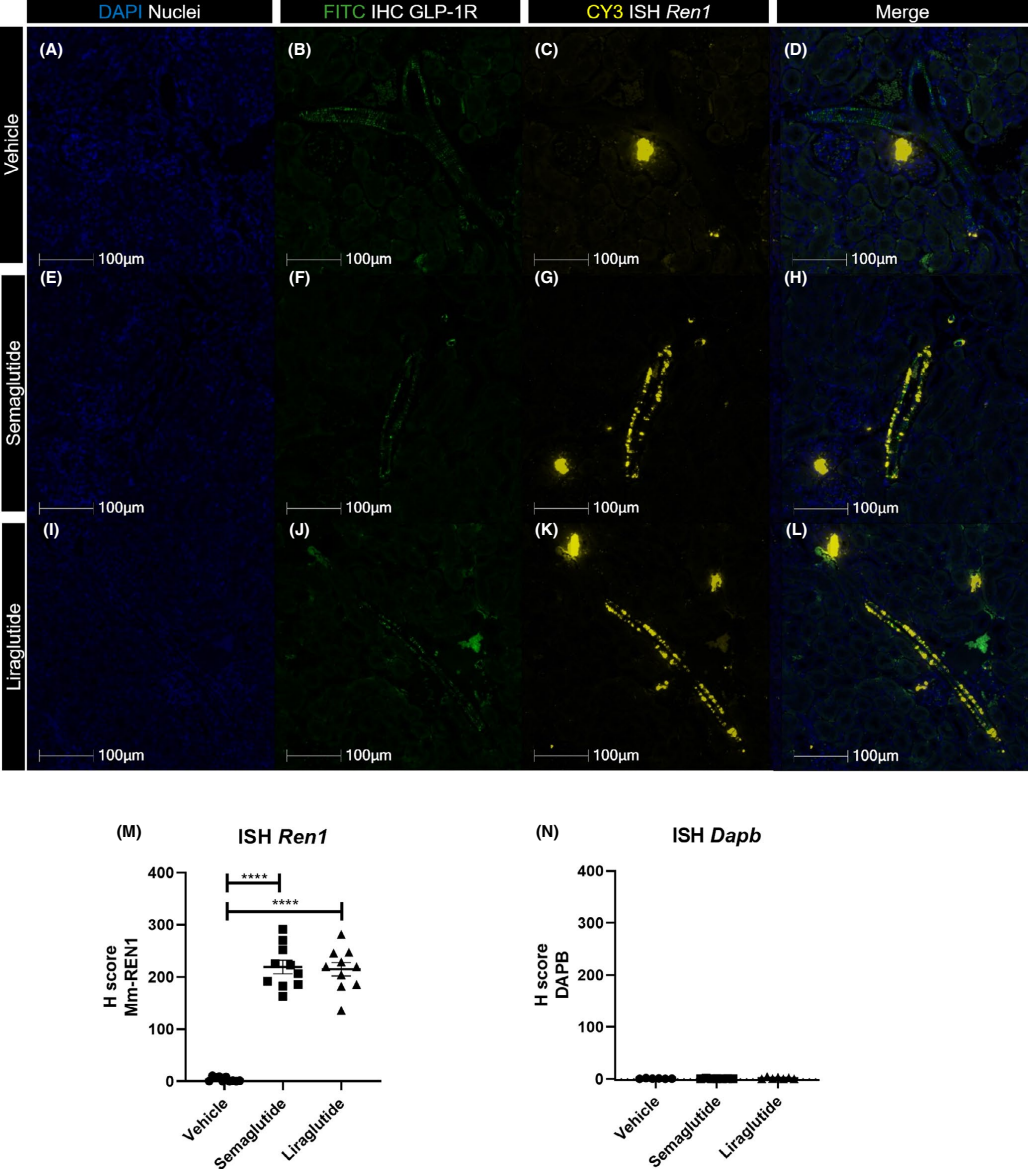
Expression of *Ren1* four hours post single injection in mouse kidney. (A‐L) Panel showing representative photos from *Ren1* in situ hybridization and GLP‐1R IHC double stain, from C57BL/6 J mice injected with a single dose of vehicle (top row), semaglutide (second row) or liraglutide (third row). DAPI nuclear stain (blue, first column), GLP‐1R IHC stain labelled with FITC (green, second column), *Ren1* ISH labelled with CY3 (yellow, column three), and merge of all three stains (Last column). Scale bars 100 µm, 40× magnification. (M) Quantification of Mm‐Ren1 detection in renal vasculature from mice injected with vehicle (●), semaglutide (■) or liraglutide (▲). Individual values represent mean of 6–9 arteries. Data shown as individual values, mean and SEM. Analysed by Brown‐Forsyth and Welch ANOVA followed by Games‐Howell's test for multiple comparisons, ****p < .0001. (N) Dapb stain as negative control from vehicle injected (●), semaglutide injected (■) and liraglutide injected (▲) mice. Individual values represent mean of 6–9 arteries. Data shown as individual values, mean and SEM

Naïve mice injected with saline (Figure 1A–D) showed expression of *Ren1* mRNA in proximity to glomeruli, with no positive stain found in the large and small blood vessels. Immunostaining using a specific anti‐GLP‐1R antibody detected GLP‐1R in arteries and arterioles only. Four hours after injection of semaglutide (Figure 1E–H) or liraglutide (Figure 1I–L), *Ren1* expression was detected in the vasculature where it co‐localized with the immunoreactivity for GLP‐1R. The levels of *Ren1* mRNA were significantly upregulated in both semaglutide and liraglutide treated animals as compared with the vehicle treated animals (*p* < .0001 for both compounds, Figure 1M).

### 3.2 Ren1 expression in kidneys from 26 days liraglutide treated NTN mice

**FIGURE 2:**
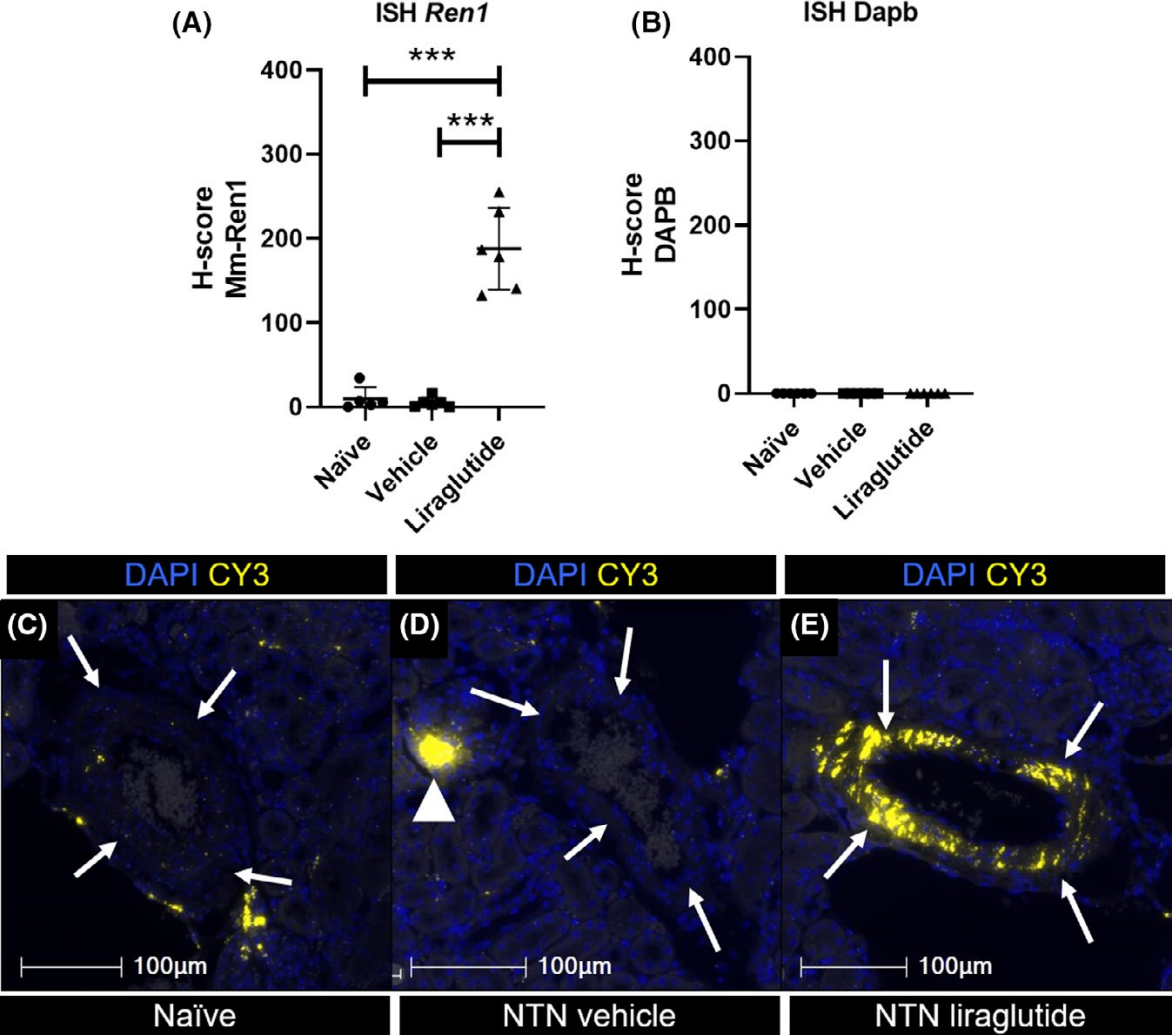
*Ren1* ISH from repeatedly injected mice. (A) Quantification of ISH of *Ren1* from Naïve mice with no treatment injection (●), vehicle treated NTN mice (■) and NTN mice treated with liraglutide for 26 days (▲). Individual values represent the mean of 5–6 selected arteries. Data shown as individual values, mean and SEM ***p ≤ .005. Brown‐Forsythe and Welch ANOVA test with Holm‐Sidak's multiple comparisons test. (B) Negative control of ISH using DAPB from naïve (●), vehicle treated NTN mice (■) and NTN mice treated with liraglutide for 26 days (▲). (C–E) Representative images of artery (white arrows) and *Ren1* in macula densa (white arrow‐head) from naïve (C), NTN dosed vehicle treated animals (D) and NTN dosed liraglutide treated animals (E). ISH of *Ren1* in CY3 (yellow) merged with nuclear stain DAPI (blue). 40× magnification, scale bar: 100 µm. NTN, nephrotoxic nephritis

ISH for *Ren1* after 26 days dosing with liraglutide showed a significant increase of *Ren1* mRNA expression as compared with naïve mice (*p* = .0004) and NTN vehicle treated mice (*p* = .0005, Figure 2A). Comparison of arteries from vehicle treated NTN mice and naïve mice showed that NTN induction *per se* did not affect *Ren1* mRNA expression in arteries (Figure 2A, C, D). A clear signal of positive *Ren1* hybridization was detected in the arteries of liraglutide treated animals (Figure 2E).

## 4 DISCUSSION

In the present study, we show that GLP‐1R activation by liraglutide or semaglutide leads to expression of the renin gene in vascular smooth muscle cells in the arteriolar and arterial part of the renal vasculature. The switch of VSMC in the renal vasculature from contractile to renin producing cells is a well‐described phenomenon known as renin recruitment.^20^

The *Ren1* gene (in humans the *REN* gene) codes for the precursor pro‐renin. The level of active renin in plasma is dependent on bioactivation by proteolytic and non‐proteolytic cleavage of the pro‐part from renin by binding to a soluble or membranous pro‐renin receptor.^21^, ^22^ The expression of renin is regulated by the levels of angiotensin II and renal perfusion pressure ^23^ and may be affected by physiological and pathological conditions including gender and diabetes mellitus.^24^ Consequently, the levels of renin mRNA expression cannot be directly translated into the amount of active renin in plasma.^25^ Published results from human studies of plasma renin levels after treatment with either native GLP‐1 or GLP‐1RA are inconsistent. In randomized placebo controlled blinded cross‐over studies of native GLP‐1 infusion in healthy men, one study showed that there was no effect of native GLP‐1 infusion on renin plasma levels in healthy volunteers,^10^ whereas another showed significantly decreased renin plasma levels.^26^ In a similar setup, there were no acute effects on renin levels in men with T2DM,^11^, ^27^ however, in a placebo controlled experimental setup in healthy subjects with high sodium intake, native GLP‐1 infusion resulted in unchanged renin plasma levels, but showed significantly reduced angiotensin II levels by 20%.^28^ These studies suggest that renin gene expression and plasma renin activity are both regulated via complex mechanisms, and the present data warrant further investigation of the transcriptional and post‐translational mechanisms that determine local and systemic renin activity following GLP‐1RA exposure.

**FIGURE 3:**
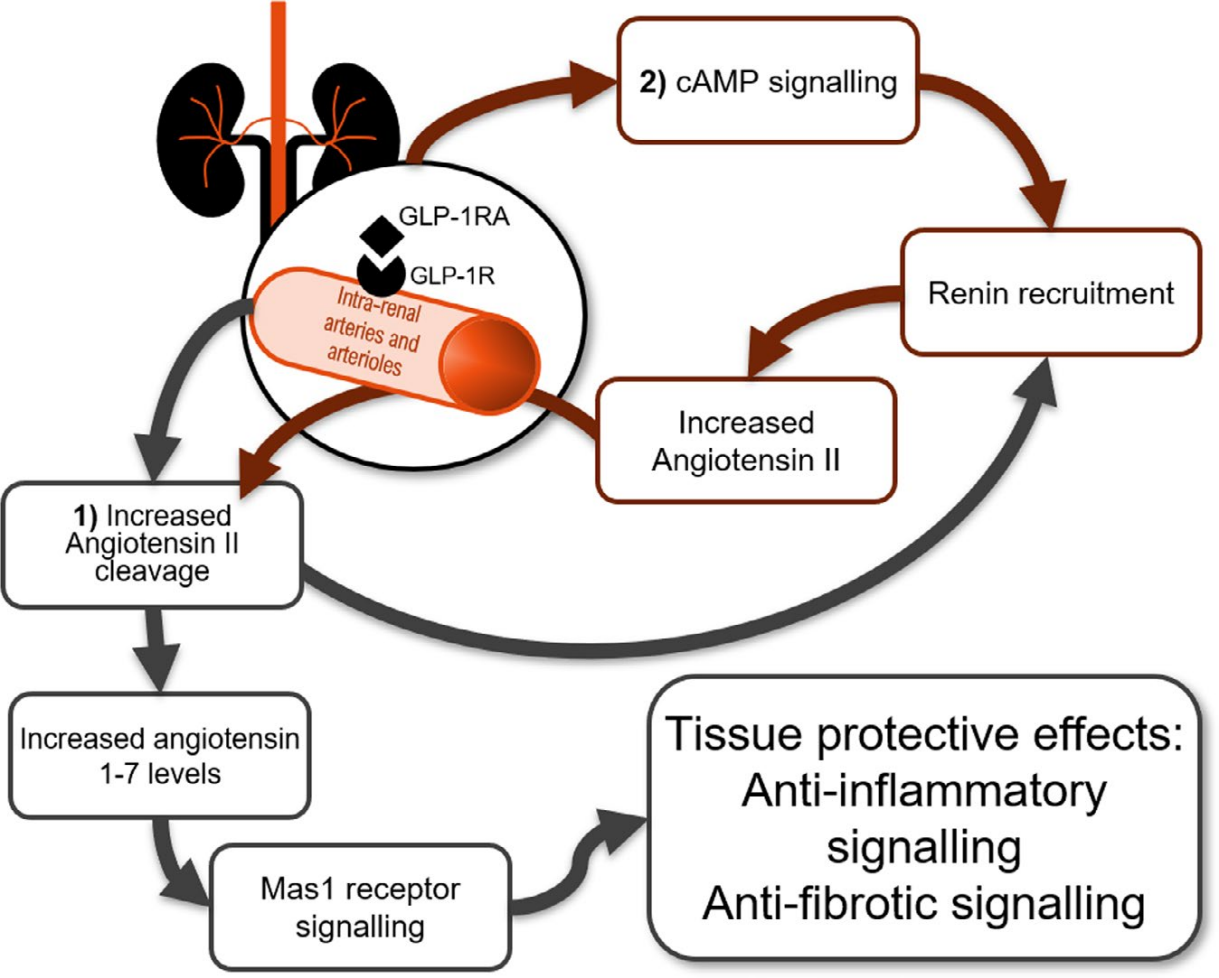
Proposed link between renal GLP‐1R activation and renin recruitment. Expression of *Ren1* after GLP‐1RA exposure may be initiated by two mechanisms, both starting with GLP‐1R activation in renal arteries: (1) Activation leads to increased angiotensin II cleavage, which in turn leads to renin recruitment by lack of negative feedback mechanism. The product of the cleavage of angiotensin II, angiotensin 1‐7, activates the Mas1 receptor resulting in anti‐inflammatory and antifibrotic effects. (2) Activation leads to an increase in cAMP levels resulting in increased expression of *Ren1* in the VSMC of the arteries. This would lead to an increase in local RAAS components including angiotensin II, which when cleaved as proposed above would further contribute to increased angiotensin 1‐7 levels

The molecular pathway leading to renin recruitment by GLP‐1R activation may include cAMP as a downstream mediator. The GLP‐1R typically activates cAMP by coupling with adenylate cyclase ^29^ and forskolin, a cAMP activator, is known to induce renin recruitment.^30^, ^31^, ^32^ Other downstream mediators of the renin recruitment effect may be factors belonging to the RAAS system. From preclinical and clinical studies RAAS inhibitors are known to decrease fibrosis, inflammation and albuminuria,^33^, ^34^, ^35^ but they also lead to renin recruitment.^36^, ^37^ In this context, GLP‐1RA’s have previously shown effects on the regulation of components of RAAS independently of haemodynamic effects. Skov et al. and Asmar et al. found that liraglutide decreased angiotensin II levels acutely in men.^10^, ^11^, ^28^ Liraglutide treatment in the NTN mouse model significantly increased *Mas1* expression in glomeruli. The *Mas1* gene codes for the receptor of angiotensin 1‐7, the peptide arising from angiotensin II following proteolytic processing by angiotensin converting enzyme 2. Hence, it could be speculated that liraglutide treatment can act locally in the kidney to induce angiotensin II cleavage and hence upregulation of the angiotensin 1‐7/mas1 pathway.^33^ This explanation is supported by an increase in angiotensin 1‐7 levels found in diabetic rats following liraglutide treatment.^38^ Angiotensin 1‐7 activation of the g‐protein coupled receptor Mas1 mediate tissue protective processes including anti‐inflammatory signalling, reduction in fibrosis, and increased endothelial signalling.^10^, ^39^ In people with diabetes, angiotensin 1‐7 levels were positively correlated with increased left ventricular ejection fraction ^40^ pointing to a positive cardiovascular effect of activation of the angiotensin 1‐7 axis (Figure 3).

Renin production leads to an increase in mean arterial pressure,^41^ and thus it is expected that an induction of renin production would lead to hypertension. Increased blood pressure is associated with increased risk of kidney damage and cardiovascular disease, which challenges the explanation for a positive effect of renin production following GLP‐1RA treatment. Nonetheless, chronic GLP‐RA treatment results in a slight decrease in systolic blood pressure and lowers the risks of cardiovascular disease and chronic kidney disease.^2^, ^3^, ^5^ This suggests that renin gene expression can stimulate biological events beyond RAAS, potentially including angiogenesis and glomerulus repair,^42^ which are beneficial for maintaining a healthy kidney.

The present study was limited to healthy mice and mice with nephrotoxic serum nephritis independent of the pathophysiological consequences of diabetic nephropathy, that includes hyperglycemia and hypoinsulinemia, conditions that are known to affect renal vascular injury.^43^ Studies in the hypercholesterolemic and prediabetic *Ldlr* ^−/−^ mouse on western diet and the NTN mouse model of chronic kidney disease showed a decrease in GLP‐1R expression in the renal vasculature in both models.^44^ This suggests that the renal vascular GLP‐1 system is affected by prediabetes and atherosclerosis as well as direct chronic kidney damage. In humans, diabetic nephropathy occurs predominantly in T2DM patients and is coincident with cardiovascular disease including atherosclerosis and hypertension.^45^ Mann et al^5^ show that liraglutide treatment significantly decreased the risk of renal disease in T2DM patients with an effect greater than the composite effect of alleviating T2DM and obesity, and the authors suggest a multifactorial mechanism including anti‐inflammatory effects. The present results suggest an involvement of renin gene expression in the NTN mouse independent of T2DM, chronic hypertension, and atherosclerosis, pointing to potential beneficial effects of GLP‐1R agonist treatment relevant for several forms of nephropathy.

In conclusion, we show that GLP‐1R activation leads to significant and robust renin gene expression in the renal vascular tree in mice and suggest that effects of renin independent of the blood pressure regulating effects of RAAS is important for the physiological and pharmacological effects of GLP‐1 on renal vascular health.

## AUTHOR CONTRIBUTION

CP and LBK conceived the presented idea and designed and oversaw the execution of experiments. KDB analyzed the results and drafted the manuscript. GFS and MEO aided in writing the manuscript and in the interpretation of data. The final paper was prepared with input from all authors.

## CONFLICT OF INTEREST

MEO, LBK and CP are employed by Novo Nordisk A/S, which markets liraglutide and semaglutide for the treatment of diabetes and weight management. LBK and CP hold minor stock portions as part of an employee‐offering programme.

## Data Availability

The data that support the findings of this study are available from the corresponding author upon reasonable request.
